# Preventing Hearing Loss and Restoring Hearing: A New Outlook

**DOI:** 10.1155/2015/868716

**Published:** 2015-03-25

**Authors:** Srdjan M. Vlajkovic, Peter R. Thorne, Ramesh Rajan, Jonathan E. Gale

**Affiliations:** ^1^Department of Physiology and Centre for Brain Research, Faculty of Medical and Health Sciences, The University of Auckland, Private Bag 92019, Auckland 1142, New Zealand; ^2^Department of Physiology, Faculty of Medicine, Nursing and Health Sciences, Monash University, Clayton, Melbourne, VIC 3800, Australia; ^3^The Ear Institute, University College London, London WCIX BEE, UK

People with hearing loss represent one of the largest disability groups worldwide, and the prevalence of hearing loss is predicted to rise with an ageing population. Substantial progress has been made towards understanding some of the biological processes involved in the development of hearing impairment as well as therapeutic ways to prevent or mitigate the hearing loss. For example, we now have a better understanding of the maintenance and regeneration of the sensorineural tissues in the cochlea. The sensory hair cells in the cochlea can potentially be regenerated by reactivating genes that control their development, and various types of stem cells can be transformed into sensory hair cells or auditory neurones. In addition, drugs that reduce oxidative stress or prevent apoptosis have been shown to protect hearing from excessive noise and ototoxic drugs. Furthermore, our knowledge of central auditory changes associated with the peripheral injury or auditory processing problems is rapidly increasing.

In this special issue, we collected review and original research articles presenting current concepts in development, screening, prevention, rehabilitation, and therapeutic management of hearing loss. It is our wish to increase interest in hearing loss research with this special issue and further accelerate the development of novel therapies for hearing disorders.

Several papers are concerned with the therapeutic management of sensorineural hearing loss, including pharmacological and cell replacement therapies. Other papers deal with the mechanisms, biomarkers, early detection, and auditory plasticity in hearing loss. Finally, one paper analyses the risk factors for noise-induced tinnitus.

The review paper by S. Irving and colleagues (Bionics Institute, University of Melbourne) presents the benefits of electroacoustic stimulation to cochlear implant recipients with residual hearing. This review also outlines the experimental and clinical strategies aimed at promoting the survival of sensory hair cells and spiral ganglion neurones in cochlear implant recipients.

Auditory nerve regeneration by human neural precursor cells (HNPC) was demonstrated by Y. Jiao et al. The study shows that BDNF supplementation improves the survival of HNPC and their migration into the brainstem in the vicinity of the cochlear nucleus. However, functional improvement after implantation of HNPC is yet to be confirmed.

Pharmacological treatment of acute noise-induced hearing loss (NIHL) is a relatively novel research field. S. M. Vlajkovic et al. report that adenosine amine congener (ADAC), a selective A_1_ adenosine receptor agonist, mitigates NIHL in a dose- and time-dependent manner. ADAC thus holds potential as a clinical treatment for noise-induced cochlear injury.

Ototoxicity is a significant impediment to anticancer treatment by cisplatin; hence different experimental and clinical strategies for prevention of cisplatin ototoxicity are reviewed by F. Chirtes and S. Albu. Gene therapies with antioxidant enzymes, neurotrophic factors, and antiapoptotic molecules combined with the intratympanic route of administration are proposed as the most promising future therapeutic options.

Understanding speech in background noise poses significant demands on cognitive resources. M. Rudner and T. Lunner in their review paper analyse cognitive spare capacity (CSC) in hearing impaired individuals and suggest ways to improve CSC with cognitive training and optimal balance of visual, phonological, and semantic information.

There is a strong need for fast and effective programs of hearing screening and surveillance. While tools exist in English, there is often a paucity of validated instruments in other languages. N. Vaez and colleagues describe the development of the Speech Understanding in Noise Test in Portuguese language, which can be used to identify individuals with hearing impairment and refer them for further audiological assessment. This provides a template for development of such tests in other languages as necessary.

According to D. Bakhos et al., children with bilateral sensorineural hearing loss and language impairment exhibit atypical temporal cortical auditory evoked potentials (CAEP), not present in hearing impaired children with normal language development. The authors propose that CAEP temporal responses can be used as a biomarker of auditory cortex maturation in children with congenital hearing loss.

The development of spontaneous activity in the auditory system prior to the onset of hearing depends on a transient cochlear structure known as Kölliker's organ. The review article by M. W. N. Dayaratne et al. describes the role of purinergic signalling in this process and how the developmental abnormalities of Kölliker's organ may lead to congenital hearing loss.

Oxidative stress is one of the principal mechanisms of sensorineural hearing loss caused by noise, ototoxic drugs, and ageing process. Excessive production of reactive oxygen species (ROS) in mitochondria reduces the antioxidant defence mechanisms and activates cell death pathways in cochlear tissues. Oxidative stress pathways leading to cell death and hearing loss are succinctly reviewed by T. Kamogashira et al. in this special issue.

Y. Zheng et al. demonstrate that acute sleep disruption does not exacerbate the perception of noise-induced tinnitus in rats, suggesting that insomnia may not be a significant risk factor/predictor for tinnitus severity as previously assumed.

We hope that this special issue will serve as a useful resource for auditory neuroscientists and clinicians and all those who have a keen interest in this research field.

